# (*N*-*sec*-Butyl-*N*-*n*-propyl­dithio­carbamato-κ^2^ *S*,*S*′)triphenyl­tin(IV)

**DOI:** 10.1107/S1600536810033039

**Published:** 2010-08-21

**Authors:** Normah Awang, Ibrahim Baba, Bohari M. Yamin, Seik Weng Ng, Edward R. T. Tiekink

**Affiliations:** a School of Chemical Sciences and Food Technology, Faculty of Science and Technology, Universiti Kebangbaan Malaysia, 43600 Bangi, Malaysia; b Department of Chemistry, University of Malaya, 50603 Kuala Lumpur, Malaysia

## Abstract

The Sn atom in the title compound, [Sn(C_6_H_5_)_3_(C_8_H_16_NS_2_)], is penta­coordinated by two S atoms, derived from an asymmetrically coordinating dithio­carbamate ligand, and three *ipso*-C atoms. The coordination geometry is inter­mediate between square-pyramidal and trigonal-bipyramidal, with a leaning towards the latter. The presence of close intra­molecular C—H⋯S contacts preclude the S atoms from forming significant inter­molecular inter­actions. Rather, mol­ecules are consolid­ated in the crystal structure by C—H⋯π inter­actions.

## Related literature

For a review of the applications and structural chemistry of tin dithio­carbamates, see: Tiekink (2008 ▶). For a related organotin structure having the same dithio­carbamate ligand, see: Abdul Muthalib *et al.* (2010 ▶). For additional structural analysis, see: Addison *et al.* (1984 ▶).
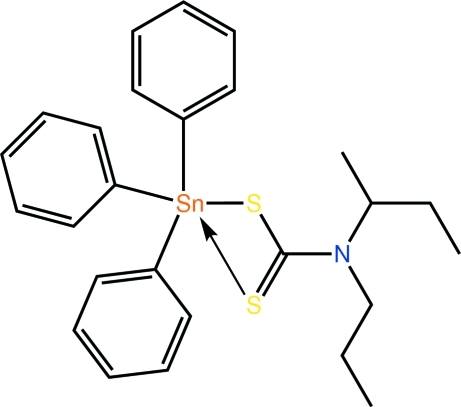


## Experimental

### 

#### Crystal data

[Sn(C_6_H_5_)_3_(C_8_H_16_NS_2_)]
*M* *_r_* = 540.33
Monoclinic, 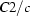

*a* = 14.7997 (5) Å
*b* = 12.1844 (5) Å
*c* = 28.8891 (11) Å
β = 97.348 (1)°
*V* = 5166.7 (3) Å^3^
*Z* = 8
Mo *K*α radiation
μ = 1.16 mm^−1^
*T* = 293 K
0.40 × 0.30 × 0.20 mm

#### Data collection

Bruker SMART diffractometer
Absorption correction: multi-scan (*SADABS*; Sheldrick, 1996 ▶) *T*_min_ = 0.653, *T*_max_ = 0.801
17218 measured reflections
5923 independent reflections
5179 reflections with *I* > 2σ(*I*)
*R*_int_ = 0.020

#### Refinement

*R*[*F* ^2^ > 2σ(*F* ^2^)] = 0.032
*wR*(*F* ^2^) = 0.083
*S* = 1.02
5923 reflections
271 parameters
H-atom parameters constrained
Δρ_max_ = 0.70 e Å^−3^
Δρ_min_ = −0.30 e Å^−3^

### 

Data collection: *SMART* (Bruker, 2002 ▶); cell refinement: *SAINT* (Bruker, 2002 ▶); data reduction: *SAINT*; program(s) used to solve structure: *SHELXS97* (Sheldrick, 2008 ▶); program(s) used to refine structure: *SHELXL97* (Sheldrick, 2008 ▶) and *PLATON* (Spek, 2009 ▶); molecular graphics: *ORTEP-3* (Farrugia, 1997 ▶) and *DIAMOND* (Brandenburg, 2006 ▶); software used to prepare material for publication: *publCIF* (Westrip, 2010 ▶).

## Supplementary Material

Crystal structure: contains datablocks global, I. DOI: 10.1107/S1600536810033039/hg2701sup1.cif

Structure factors: contains datablocks I. DOI: 10.1107/S1600536810033039/hg2701Isup2.hkl

Additional supplementary materials:  crystallographic information; 3D view; checkCIF report

## Figures and Tables

**Table 1:** Hydrogen-bond geometry (Å, °) *Cg*1 and *Cg*2 are the centroids of the C1–C6 and C7–C12 rings, respectively.

*D*—H⋯*A*	*D*—H	H⋯*A*	*D*⋯*A*	*D*—H⋯*A*
C14—H14⋯S2	0.93	2.75	3.433 (3)	131
C20—H20⋯S2	0.98	2.49	3.059 (3)	117
C24—H24a⋯S1	0.97	2.58	2.938 (3)	102
C25—H25b⋯S1	0.97	2.84	3.360 (4)	115
C16—H16⋯*Cg*1^i^	0.93	2.78	3.618 (3)	151
C23—H23a⋯*Cg*2^ii^	0.96	2.91	3.773 (4)	150

Symmetry codes: (i) 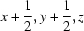; (ii) 
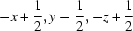
.

**Fig. 1.**
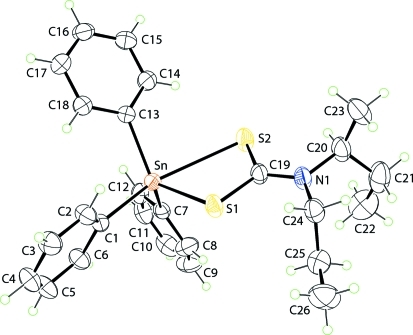
The molecular structure of (I) showing the atom-labelling scheme and displacement ellipsoids at the 50% probability level.

**Fig. 2.**
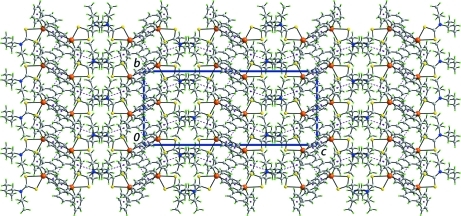
A view in projection down the a axis of (I) showing the unit-cell contents. The C–H···π contacts are shown as purple dashed lines.

## supplementary crystallographic information

### Comment

Organotin dithiocarbamates display properties that suggest their use as anti-cancer agents, anti-microbial agents and as insecticides (Tiekink, 2008). Such interest motivates on-going structural characterization of such compounds (Abdul Muthalib *et al.*, 2010) and led to the investigation of the title compound, (I).

The Sn atom in (I) is penta-coordinated by two S atoms derived from an asymmetrically coordinating dithiocarbamate ligand and three *ipso*-C atoms from the phenyl substituents, Fig. 1. The resulting C_3_S_2_ coordination geometry is intermediate between square pyramidal and trigonal bi-pyramidal with a leaning towards the latter. Thus, compared to the ideal values for τ of 0.0 and 1.0 for ideal square pyramidal and trigonal bi-pyramidal geometries, respectively (Addison *et al.*, 1984), the value for τ in (I) computes to 0.55. The asymmetric mode of coordination of the dithiocarbamate ligand is reflected in significant differences in the associated C–S bond distances with that formed by the S1 atom, involved in the shorter of the Sn–S bonds, being considerably longer [S1–C19 = 1.755 (3) Å] than that formed by the S2 atom [S2–C19 = 1.682 (3) Å]. The observed molecular structure is entirely consistent with literature precedents (Tiekink, 2008).

Each of the S atoms is involved in two intramolecular C–H···S contacts and these do not participate in intermolecular interactions, Table 2. The presence of C–H···π contacts are noted, Table 1, and occur between benzene- and methyl-H atoms with two of the Sn-bound benzene rings functioning as the acceptors. These serve to consolidate the molecules into the crystal structure, Fig. 2.

### Experimental

Carbon disulfide (30 mmol) was dropped into an ethanol solution (100 ml) of *N*-*sec*-butyl-*N*-*n*-propylamine (30 mmol). The solution was kept at 273 K for an hour. Triphenyltin chloride (30 mmol) dissolved in ethanol (100 ml) was added to give a white precipitate. This was collected and colourless crystals were obtained by recrystallization from its chloroform/ethanol (1/1) mixture.

### Refinement

Carbon-bound H-atoms were placed in calculated positions (C—H 0.93 to 0.98 Å) and were included in the refinement in the riding model approximation, with *U*_iso_(H) set to 1.2 to 1.5*U*_equiv_(C).

### Crystal data

**Table:**

[Sn(C_6_H_5_)_3_(C_8_H_16_NS_2_)]	*F*(000) = 2208
*M**_r_* = 540.33	*D*_x_ = 1.389 Mg m^−^^3^
Monoclinic, *C*2/*c*	Mo *K*α radiation, λ = 0.71073 Å
Hall symbol: -C 2yc	Cell parameters from 7436 reflections
*a* = 14.7997 (5) Å	θ = 2.1–27.2°
*b* = 12.1844 (5) Å	µ = 1.16 mm^−^^1^
*c* = 28.8891 (11) Å	*T* = 293 K
β = 97.348 (1)°	Block, colourless
*V* = 5166.7 (3) Å^3^	0.40 × 0.30 × 0.20 mm
*Z* = 8	

### Data collection

**Table:**

Bruker SMART diffractometer	5923 independent reflections
Radiation source: fine-focus sealed tube	5179 reflections with *I* > 2σ(*I*)
graphite	*R*_int_ = 0.020
ω scans	θ_max_ = 27.5°, θ_min_ = 1.4°
Absorption correction: multi-scan (*SADABS*; Sheldrick, 1996)	*h* = −19→17
*T*_min_ = 0.653, *T*_max_ = 0.801	*k* = −14→15
17218 measured reflections	*l* = −34→37

### Refinement

**Table:**

Refinement on *F*^2^	Primary atom site location: structure-invariant direct methods
Least-squares matrix: full	Secondary atom site location: difference Fourier map
*R*[*F*^2^ > 2σ(*F*^2^)] = 0.032	Hydrogen site location: inferred from neighbouring sites
*wR*(*F*^2^) = 0.083	H-atom parameters constrained
*S* = 1.02	*w* = 1/[σ^2^(*F*_o_^2^) + (0.0437*P*)^2^ + 4.4496*P*] where *P* = (*F*_o_^2^ + 2*F*_c_^2^)/3
5923 reflections	(Δ/σ)_max_ < 0.001
271 parameters	Δρ_max_ = 0.70 e Å^−^^3^
0 restraints	Δρ_min_ = −0.30 e Å^−^^3^

### Fractional atomic coordinates and isotropic or equivalent isotropic displacement parameters (Å^2^)

**Table:**

	*x*	*y*	*z*	*U*_iso_*/*U*_eq_	
Sn	0.247917 (10)	0.577523 (13)	0.418770 (5)	0.04008 (7)	
S1	0.22291 (6)	0.39926 (6)	0.37826 (2)	0.05486 (18)	
S2	0.28766 (6)	0.56989 (6)	0.31823 (2)	0.0588 (2)	
C1	0.21407 (15)	0.50813 (19)	0.48322 (8)	0.0397 (5)	
C2	0.26523 (19)	0.4242 (2)	0.50565 (10)	0.0520 (6)	
H2	0.3156	0.3979	0.4929	0.062*	
C3	0.2432 (2)	0.3786 (3)	0.54658 (11)	0.0616 (7)	
H3	0.2786	0.3222	0.5611	0.074*	
C4	0.1697 (2)	0.4162 (3)	0.56561 (11)	0.0673 (9)	
H4	0.1551	0.3861	0.5933	0.081*	
C5	0.1171 (2)	0.4987 (3)	0.54404 (11)	0.0704 (9)	
H5	0.0662	0.5235	0.5568	0.085*	
C6	0.13936 (19)	0.5452 (2)	0.50331 (10)	0.0542 (6)	
H6	0.1039	0.6019	0.4892	0.065*	
C7	0.14476 (16)	0.6950 (2)	0.39602 (8)	0.0445 (5)	
C8	0.06015 (19)	0.6649 (3)	0.37279 (11)	0.0649 (8)	
H8	0.0484	0.5916	0.3654	0.078*	
C9	−0.0062 (2)	0.7423 (4)	0.36066 (13)	0.0811 (11)	
H9	−0.0623	0.7209	0.3450	0.097*	
C10	0.0095 (2)	0.8508 (4)	0.37143 (12)	0.0774 (10)	
H10	−0.0357	0.9028	0.3631	0.093*	
C11	0.0915 (3)	0.8821 (3)	0.39429 (11)	0.0696 (9)	
H11	0.1021	0.9556	0.4019	0.084*	
C12	0.1597 (2)	0.8046 (2)	0.40645 (9)	0.0541 (6)	
H12	0.2158	0.8270	0.4217	0.065*	
C13	0.38064 (15)	0.6470 (2)	0.43524 (8)	0.0419 (5)	
C14	0.42367 (19)	0.7084 (3)	0.40417 (10)	0.0585 (7)	
H14	0.3981	0.7127	0.3731	0.070*	
C15	0.5041 (2)	0.7634 (3)	0.41863 (11)	0.0650 (8)	
H15	0.5320	0.8042	0.3973	0.078*	
C16	0.54271 (18)	0.7581 (3)	0.46412 (11)	0.0593 (7)	
H16	0.5967	0.7954	0.4737	0.071*	
C17	0.50182 (19)	0.6978 (3)	0.49562 (11)	0.0599 (7)	
H17	0.5279	0.6940	0.5266	0.072*	
C18	0.42128 (18)	0.6423 (2)	0.48114 (9)	0.0523 (6)	
H18	0.3940	0.6011	0.5027	0.063*	
C19	0.24957 (19)	0.4410 (2)	0.32344 (9)	0.0473 (6)	
C20	0.2709 (3)	0.3917 (3)	0.24303 (10)	0.0678 (9)	
H20	0.2875	0.4696	0.2433	0.081*	
C21	0.1982 (3)	0.3750 (4)	0.20404 (12)	0.0915 (12)	
H21A	0.1746	0.3010	0.2054	0.110*	
H21B	0.2233	0.3830	0.1748	0.110*	
C22	0.1203 (3)	0.4571 (4)	0.20540 (17)	0.1028 (14)	
H22A	0.0741	0.4437	0.1796	0.154*	
H22B	0.1433	0.5304	0.2034	0.154*	
H22C	0.0948	0.4487	0.2341	0.154*	
C23	0.3572 (3)	0.3250 (3)	0.23634 (13)	0.0822 (11)	
H23A	0.3761	0.3427	0.2067	0.123*	
H23B	0.3440	0.2480	0.2374	0.123*	
H23C	0.4052	0.3430	0.2608	0.123*	
C24	0.2103 (2)	0.2516 (2)	0.29729 (10)	0.0591 (7)	
H24A	0.2382	0.2270	0.3277	0.071*	
H24B	0.2312	0.2036	0.2741	0.071*	
C25	0.1091 (2)	0.2416 (3)	0.29490 (12)	0.0711 (8)	
H25A	0.0810	0.2550	0.2632	0.085*	
H25B	0.0865	0.2962	0.3150	0.085*	
C27	0.0838 (3)	0.1271 (3)	0.31025 (17)	0.0987 (13)	
H27A	0.0188	0.1211	0.3082	0.148*	
H27B	0.1105	0.1148	0.3419	0.148*	
H27C	0.1063	0.0733	0.2903	0.148*	
N1	0.24161 (17)	0.36613 (19)	0.28932 (7)	0.0540 (5)	

### Atomic displacement parameters (Å^2^)

**Table:**

	*U*^11^	*U*^22^	*U*^33^	*U*^12^	*U*^13^	*U*^23^
Sn	0.04146 (10)	0.04203 (11)	0.03675 (10)	−0.00450 (7)	0.00505 (7)	0.00138 (6)
S1	0.0814 (5)	0.0478 (4)	0.0381 (3)	−0.0154 (3)	0.0179 (3)	−0.0017 (3)
S2	0.0903 (5)	0.0459 (4)	0.0421 (4)	−0.0182 (3)	0.0164 (3)	−0.0020 (3)
C1	0.0419 (11)	0.0412 (12)	0.0364 (11)	−0.0039 (10)	0.0066 (9)	−0.0017 (9)
C2	0.0474 (14)	0.0594 (17)	0.0509 (15)	0.0071 (12)	0.0129 (12)	0.0080 (12)
C3	0.0612 (17)	0.0668 (19)	0.0568 (17)	0.0063 (15)	0.0070 (13)	0.0203 (14)
C4	0.080 (2)	0.080 (2)	0.0455 (16)	−0.0051 (17)	0.0205 (15)	0.0131 (14)
C5	0.0710 (19)	0.083 (2)	0.0644 (19)	0.0102 (17)	0.0366 (15)	0.0018 (17)
C6	0.0553 (15)	0.0532 (15)	0.0566 (16)	0.0098 (12)	0.0164 (12)	0.0027 (12)
C7	0.0437 (12)	0.0550 (15)	0.0355 (12)	−0.0011 (11)	0.0074 (9)	0.0085 (10)
C8	0.0526 (15)	0.069 (2)	0.0700 (19)	−0.0120 (14)	−0.0053 (14)	0.0156 (15)
C9	0.0464 (16)	0.109 (3)	0.084 (2)	−0.0040 (18)	−0.0042 (15)	0.035 (2)
C10	0.070 (2)	0.097 (3)	0.068 (2)	0.031 (2)	0.0197 (17)	0.0336 (19)
C11	0.096 (2)	0.0607 (19)	0.0541 (17)	0.0186 (18)	0.0150 (17)	0.0078 (14)
C12	0.0634 (16)	0.0553 (16)	0.0427 (14)	0.0011 (13)	0.0036 (12)	0.0021 (11)
C13	0.0386 (11)	0.0462 (13)	0.0415 (12)	−0.0025 (10)	0.0073 (9)	−0.0015 (10)
C14	0.0583 (16)	0.072 (2)	0.0449 (14)	−0.0156 (14)	0.0058 (12)	0.0049 (13)
C15	0.0580 (17)	0.076 (2)	0.0632 (18)	−0.0208 (15)	0.0179 (14)	0.0008 (15)
C16	0.0394 (13)	0.0664 (19)	0.0721 (19)	−0.0072 (12)	0.0073 (12)	−0.0128 (15)
C17	0.0488 (14)	0.075 (2)	0.0538 (16)	−0.0035 (14)	−0.0026 (12)	−0.0035 (14)
C18	0.0491 (14)	0.0628 (17)	0.0451 (14)	−0.0060 (12)	0.0059 (11)	0.0030 (12)
C19	0.0597 (15)	0.0479 (14)	0.0350 (12)	−0.0078 (11)	0.0089 (11)	−0.0007 (10)
C20	0.095 (2)	0.0675 (19)	0.0435 (15)	−0.0198 (18)	0.0200 (15)	−0.0022 (13)
C21	0.140 (4)	0.084 (3)	0.0484 (18)	−0.018 (3)	0.005 (2)	0.0025 (17)
C22	0.107 (3)	0.090 (3)	0.104 (3)	0.009 (3)	−0.015 (3)	0.023 (3)
C23	0.095 (3)	0.090 (3)	0.070 (2)	0.003 (2)	0.041 (2)	−0.0058 (18)
C24	0.0743 (19)	0.0518 (16)	0.0531 (16)	0.0002 (14)	0.0152 (13)	−0.0078 (13)
C25	0.075 (2)	0.070 (2)	0.069 (2)	−0.0063 (17)	0.0095 (16)	−0.0089 (16)
C27	0.111 (3)	0.066 (2)	0.127 (4)	−0.029 (2)	0.049 (3)	−0.005 (2)
N1	0.0759 (15)	0.0499 (13)	0.0382 (11)	−0.0122 (11)	0.0150 (10)	−0.0048 (9)

### Geometric parameters (Å, °)

**Table:**

Sn—C1	2.161 (2)	C14—H14	0.9300
Sn—C7	2.134 (3)	C15—C16	1.366 (4)
Sn—C1	2.161 (2)	C15—H15	0.9300
Sn—C13	2.136 (2)	C16—C17	1.370 (4)
Sn—C1	2.161 (2)	C16—H16	0.9300
Sn—S1	2.4725 (7)	C17—C18	1.388 (4)
S1—C19	1.755 (3)	C17—H17	0.9300
S2—C19	1.682 (3)	C18—H18	0.9300
C1—C6	1.388 (3)	C19—N1	1.337 (3)
C1—C2	1.383 (3)	C20—C21	1.468 (5)
C2—C3	1.383 (4)	C20—N1	1.491 (4)
C2—H2	0.9300	C20—C23	1.548 (5)
C3—C4	1.360 (4)	C20—H20	0.9800
C3—H3	0.9300	C21—C22	1.530 (6)
C4—C5	1.371 (5)	C21—H21A	0.9700
C4—H4	0.9300	C21—H21B	0.9700
C5—C6	1.383 (4)	C22—H22A	0.9600
C5—H5	0.9300	C22—H22B	0.9600
C6—H6	0.9300	C22—H22C	0.9600
C7—C12	1.381 (4)	C23—H23A	0.9600
C7—C8	1.392 (4)	C23—H23B	0.9600
C8—C9	1.374 (5)	C23—H23C	0.9600
C8—H8	0.9300	C24—N1	1.497 (4)
C9—C10	1.371 (6)	C24—C25	1.496 (4)
C9—H9	0.9300	C24—H24A	0.9700
C10—C11	1.360 (5)	C24—H24B	0.9700
C10—H10	0.9300	C25—C27	1.526 (5)
C11—C12	1.394 (4)	C25—H25A	0.9700
C11—H11	0.9300	C25—H25B	0.9700
C12—H12	0.9300	C27—H27A	0.9600
C13—C18	1.385 (3)	C27—H27B	0.9600
C13—C14	1.385 (4)	C27—H27C	0.9600
C14—C15	1.383 (4)		
			
C7—Sn—C13	113.84 (10)	C16—C17—C18	119.8 (3)
C7—Sn—C1	106.99 (9)	C16—C17—H17	120.1
C13—Sn—C1	105.72 (9)	C18—C17—H17	120.1
C7—Sn—S1	112.67 (7)	C13—C18—C17	121.3 (3)
C13—Sn—S1	122.09 (7)	C13—C18—H18	119.4
C1—Sn—S1	91.54 (6)	C17—C18—H18	119.4
C19—S1—Sn	97.79 (9)	N1—C19—S2	124.7 (2)
C6—C1—C2	117.5 (2)	N1—C19—S1	117.3 (2)
C6—C1—Sn	121.17 (19)	S2—C19—S1	117.87 (15)
C2—C1—Sn	121.29 (18)	C21—C20—N1	113.0 (3)
C3—C2—C1	121.5 (3)	C21—C20—C23	111.5 (3)
C3—C2—H2	119.2	N1—C20—C23	110.0 (3)
C1—C2—H2	119.2	C21—C20—H20	107.4
C4—C3—C2	119.9 (3)	N1—C20—H20	107.4
C4—C3—H3	120.1	C23—C20—H20	107.4
C2—C3—H3	120.1	C20—C21—C22	111.7 (3)
C3—C4—C5	120.1 (3)	C20—C21—H21A	109.3
C3—C4—H4	120.0	C22—C21—H21A	109.3
C5—C4—H4	120.0	C20—C21—H21B	109.3
C6—C5—C4	120.2 (3)	C22—C21—H21B	109.3
C6—C5—H5	119.9	H21A—C21—H21B	107.9
C4—C5—H5	119.9	C21—C22—H22A	109.5
C5—C6—C1	120.8 (3)	C21—C22—H22B	109.5
C5—C6—H6	119.6	H22A—C22—H22B	109.5
C1—C6—H6	119.6	C21—C22—H22C	109.5
C12—C7—C8	118.0 (3)	H22A—C22—H22C	109.5
C12—C7—Sn	119.54 (19)	H22B—C22—H22C	109.5
C8—C7—Sn	122.4 (2)	C20—C23—H23A	109.5
C9—C8—C7	120.8 (3)	C20—C23—H23B	109.5
C9—C8—H8	119.6	H23A—C23—H23B	109.5
C7—C8—H8	119.6	C20—C23—H23C	109.5
C8—C9—C10	120.6 (3)	H23A—C23—H23C	109.5
C8—C9—H9	119.7	H23B—C23—H23C	109.5
C10—C9—H9	119.7	N1—C24—C25	113.3 (3)
C11—C10—C9	119.7 (3)	N1—C24—H24A	108.9
C11—C10—H10	120.1	C25—C24—H24A	108.9
C9—C10—H10	120.1	N1—C24—H24B	108.9
C10—C11—C12	120.3 (3)	C25—C24—H24B	108.9
C10—C11—H11	119.8	H24A—C24—H24B	107.7
C12—C11—H11	119.8	C24—C25—C27	110.0 (3)
C7—C12—C11	120.6 (3)	C24—C25—H25A	109.7
C7—C12—H12	119.7	C27—C25—H25A	109.7
C11—C12—H12	119.7	C24—C25—H25B	109.7
C18—C13—C14	117.6 (2)	C27—C25—H25B	109.7
C18—C13—Sn	118.10 (18)	H25A—C25—H25B	108.2
C14—C13—Sn	123.77 (18)	C25—C27—H27A	109.5
C15—C14—C13	121.0 (3)	C25—C27—H27B	109.5
C15—C14—H14	119.5	H27A—C27—H27B	109.5
C13—C14—H14	119.5	C25—C27—H27C	109.5
C16—C15—C14	120.4 (3)	H27A—C27—H27C	109.5
C16—C15—H15	119.8	H27B—C27—H27C	109.5
C14—C15—H15	119.8	C19—N1—C20	120.7 (2)
C15—C16—C17	119.9 (3)	C19—N1—C24	121.5 (2)
C15—C16—H16	120.0	C20—N1—C24	117.7 (2)
C17—C16—H16	120.0		
			
C7—Sn—S1—C19	71.53 (12)	C10—C11—C12—C7	−0.8 (5)
C13—Sn—S1—C19	−69.63 (13)	C7—Sn—C13—C18	114.8 (2)
C1—Sn—S1—C19	−179.32 (11)	C1—Sn—C13—C18	−2.4 (2)
C7—Sn—C1—C6	−2.9 (2)	S1—Sn—C13—C18	−104.5 (2)
C13—Sn—C1—C6	118.8 (2)	C7—Sn—C13—C14	−56.8 (3)
S1—Sn—C1—C6	−117.2 (2)	C1—Sn—C13—C14	−173.9 (2)
C7—Sn—C1—C2	176.2 (2)	S1—Sn—C13—C14	84.0 (2)
C13—Sn—C1—C2	−62.1 (2)	C18—C13—C14—C15	−0.3 (4)
S1—Sn—C1—C2	62.0 (2)	Sn—C13—C14—C15	171.3 (2)
C6—C1—C2—C3	0.0 (4)	C13—C14—C15—C16	0.0 (5)
Sn—C1—C2—C3	−179.2 (2)	C14—C15—C16—C17	0.2 (5)
C1—C2—C3—C4	0.0 (5)	C15—C16—C17—C18	0.0 (5)
C2—C3—C4—C5	0.6 (5)	C14—C13—C18—C17	0.5 (4)
C3—C4—C5—C6	−1.1 (5)	Sn—C13—C18—C17	−171.6 (2)
C4—C5—C6—C1	1.1 (5)	C16—C17—C18—C13	−0.4 (5)
C2—C1—C6—C5	−0.5 (4)	Sn—S1—C19—N1	−178.0 (2)
Sn—C1—C6—C5	178.6 (2)	Sn—S1—C19—S2	4.69 (18)
C13—Sn—C7—C12	−19.9 (2)	N1—C20—C21—C22	66.8 (4)
C1—Sn—C7—C12	96.5 (2)	C23—C20—C21—C22	−168.7 (3)
S1—Sn—C7—C12	−164.36 (18)	N1—C24—C25—C27	−171.8 (3)
C13—Sn—C7—C8	163.4 (2)	S2—C19—N1—C20	2.7 (4)
C1—Sn—C7—C8	−80.2 (2)	S1—C19—N1—C20	−174.4 (2)
S1—Sn—C7—C8	18.9 (2)	S2—C19—N1—C24	177.7 (2)
C12—C7—C8—C9	0.0 (4)	S1—C19—N1—C24	0.6 (4)
Sn—C7—C8—C9	176.8 (3)	C21—C20—N1—C19	−124.4 (3)
C7—C8—C9—C10	−0.3 (5)	C23—C20—N1—C19	110.2 (3)
C8—C9—C10—C11	0.0 (5)	C21—C20—N1—C24	60.4 (4)
C9—C10—C11—C12	0.5 (5)	C23—C20—N1—C24	−65.0 (4)
C8—C7—C12—C11	0.5 (4)	C25—C24—N1—C19	80.9 (3)
Sn—C7—C12—C11	−176.3 (2)	C25—C24—N1—C20	−103.9 (3)

### Hydrogen-bond geometry (Å, °)

**Table:**

Cg1 and Cg2 are the centroids of the C1–C6 and C7–C12 rings, respectively.

**Table:**

*D*—H···*A*	*D*—H	H···*A*	*D*···*A*	*D*—H···*A*
C14—H14···S2	0.93	2.75	3.433 (3)	131
C20—H20···S2	0.98	2.49	3.059 (3)	117
C24—H24a···S1	0.97	2.58	2.938 (3)	102
C25—H25b···S1	0.97	2.84	3.360 (4)	115
C16—H16···Cg1^i^	0.93	2.78	3.618 (3)	151
C23—H23a···Cg2^ii^	0.96	2.91	3.773 (4)	150

Symmetry codes:  (i) *x*+1/2, *y*+1/2, *z*; (ii) −*x*+1/2, *y*−1/2, −*z*+1/2.
